# Perceptions and Practicalities Influencing Pre-exposure Prophylaxis Adherence Among Men Who Have Sex with Men in England

**DOI:** 10.1007/s10461-022-03624-6

**Published:** 2022-02-19

**Authors:** Dora Arnold-Forster, Robert Horne, Will Nutland, Sonali Wayal, Michael Rayment, Caroline Rae, Monica Desai, Amanda Clarke, Ann Sullivan, Sheena McCormack, Mitzy Gafos

**Affiliations:** 1 Department of Global Health and Development, London School of Hygiene and Tropical Medicine, Kepple Street, London, WC1E 7HT UK; 2 School of Pharmacy, University College London, London, UK; 3 Prepster, London, UK; 4 Institute for Global Health, University College London, London, UK; 5 Chelsea and Westminster Hospital NHS Foundation Trust, London, UK; 6 National Institute for Health and Care Excellence, London, UK; 7 Claude Nicol Centre, Royal Sussex County Hospital, Brighton, UK; 8 Medical Research Council Clinical Trials Unit, Institute of Clinical Trials and Methodology, University College London, London, UK

**Keywords:** Perceptions and practicalities approach (PAPA), Pre-exposure prophylaxis (PrEP), Adherence

## Abstract

PrEP is highly effective for HIV prevention but requires adequate adherence. In this paper we use the perceptions and practicalities approach (PAPA) to identify factors that influenced PrEP adherence using qualitative data from the PROUD study. From February 2014 to January 2016, we interviewed 41 gay, bisexual and other men-who-have-sex-with-men and one trans woman who were enrolled in the study. We purposively recruited participants for interview based on trial arm allocation, adherence and sexual risk behaviours. The interviews were conducted in English, audio-recorded, transcribed, coded and analysed using framework analysis. Participants in general were highly motivated to use and adhere to PrEP, and this was linked to strong perceptions of personal necessity for PrEP as they felt at risk of HIV and viewed PrEP as highly effective. On the other hand, concerns about side effects and HIV resistance did inhibit PrEP initiation and adherence although this was uncommon. Practical factors such as daily routine, existing habitual pill-taking and pill storage impacted adherence. Drug and alcohol use rarely caused participants to miss doses. These findings indicate that using the principals of PAPA to unpick influencers of PrEP use, could help tailor adherence support in PrEP programmes.

**Supplementary Information:**

The online version contains supplementary material available at 10.1007/s10461-022-03624-6.

## Introduction

HIV in men who have sex with men (MSM) continues to be of high public health importance in the UK with 34% of the 2,630 new HIV cases in 2020 reported among MSM [1]. In 2015 the World Health Organization (WHO) recommended pre-exposure prophylaxis (PrEP) to prevent sexual acquisition of HIV as part of a combination prevention approach for high-risk groups, including transgender women (TGW) and MSM [2] based on evidence of several clinical trials that demonstrated PrEP to be highly effective when taken correctly [3–6]. This includes the UK PROUD trial, which reported a relative risk reduction of 86% in HIV incidence for those offered PrEP in the intervention arm [5]. However, inadequate adherence to either the daily or the on-demand dosing schedule compromises effectiveness [7–9]. For example, the international iPrEx PrEP trial reported a much lower protective effect of 44%, but a risk reduction of 92% was estimated for participants with detectable drug levels [6]. Adequate adherence (at least 4 pills per week for those on daily PrEP) is also vital to prevent drug resistant strains of HIV, which can emerge with continued use when one has unknowingly seroconverted [8, 10].

Efforts to improve PrEP adherence, and hence efficacy, are likely to be more effective if they are informed by theories of adherence. These can identify modifiable causes of nonadherence that can be addressed through pragmatic interventions. The perceptions and practicalities approach is a pragmatic framework for the development of adherence support [11, 12]. It begins by recognising that adherence is a variable behaviour rather than a trait characteristic. Adherence rates vary, not just between individuals, but within the same individual over time and across treatments. Most of us are nonadherent some of the time and nonadherence is often the norm rather than the exception [13]. Indeed, a meta-analysis of 569 studies found 24.8% nonadherence across multiple diseases [14].

There are a range of structural, social, economic, and psychological factors that influence adherence. For example, a systematic review and meta-synthesis of HIV treatment adherence identified stigma, negative social norms, lack of social support and coping mechanisms, poverty, and poor health care services as negatively impacting adherence [15]. Based on these factors it follows that adherence support should be tailored to the needs of the individual taking account of both the perceptions (e.g. beliefs about the illness and treatment) and practicalities (e.g. capability and resources) influencing *motivation* and *ability* to start and continue with the treatment.

To date the vast majority of HIV prevention adherence research reports on the ‘practical’ factors that influence a persons’ *ability* to adhere, but rarely report the perceptions that influence a persons’ *motivation* to adhere. The key *perceptual* beliefs influencing how an individual adheres to treatment are how they judge their personal *need* for it (necessity beliefs) relative to their *concerns* about taking it, as specified in the necessity concerns framework (NCF). [16]. The NCF has been demonstrated to be predictive of low adherence to treatment (in terms of lower belief of necessity and higher concerns about the medication) across a range of illnesses including HIV treatment [17–19]. The PAPA and NCF approach is applied in NICE guidelines for adherence [20].

The PAPA framework also acknowledges the importance of environmental and social factors (external factors) in creating the opportunity for adherence and the role of internal and external triggers (such as reminders) but posits that these have their effect by influencing individual motivation and/or ability. An essential approach to understanding and addressing variations in adherence is to consider what influences the individual to want or not want to adhere and what factors influence their ability to do so.

PrEP is now widely available through health services in many countries globally, including the UK [21]. Whilst there have been some studies regarding PrEP acceptability or hypothetical use in the UK [22, 23], the PROUD qualitative study was the first to interview PrEP users about their experiences of pill-use. While the PROUD study was designed to assess the effectiveness of daily PrEP, the IPERGAY trial was evaluating on-demand dosing with PrEP before and after sex (two tablets up to 24 h before sex, a third tablet 24 h after and fourth tablet 48 h after the initial dose). Also, a sub-analysis of the iPrEx data suggested 4 doses per week provided sufficient protection, and injectable PrEP was in development at the time of interview. As such, we also probed participants about their preferred dosing (daily, on-demand, 4-doses per week, monthly) and administration (pill, injectable) options to optimize adherence. This analysis identifies the influencers of adherence to PrEP and examines attitudes towards other (non-daily) dosing schedules and a long-acting injectable PrEP.

## Methods

### PROUD Trial Design

PROUD was an open-label waitlist design trial to evaluate the effectiveness of daily Truvada as PrEP. Recruitment took place from November 2012 to April 2014 at 13 sexual health clinics in England. Eligible participants were HIV-negative gay bisexual or other MSM (GBMSM) or TGW, who reported condomless anal sex in the last three months and anticipated it again in the next three months. Participants were randomised 1:1 to receive PrEP immediately (immediate arm) or to a deferred start after 12 months (deferred arm). PrEP was prescribed in the form of a single daily tablet containing tenofovir disproxil fumarate and emtricitabine (Truvada; Gilead Sciences, Foster City, CA, USA). However, on 13th October 2014 the trial steering committee recommended that deferred participants be offered PrEP due to early results demonstrating its effectiveness. The study design, results and the cohort’s baseline characteristics are reported elsewhere [5, 24].

### Qualitative Study

**Fig. 1:**
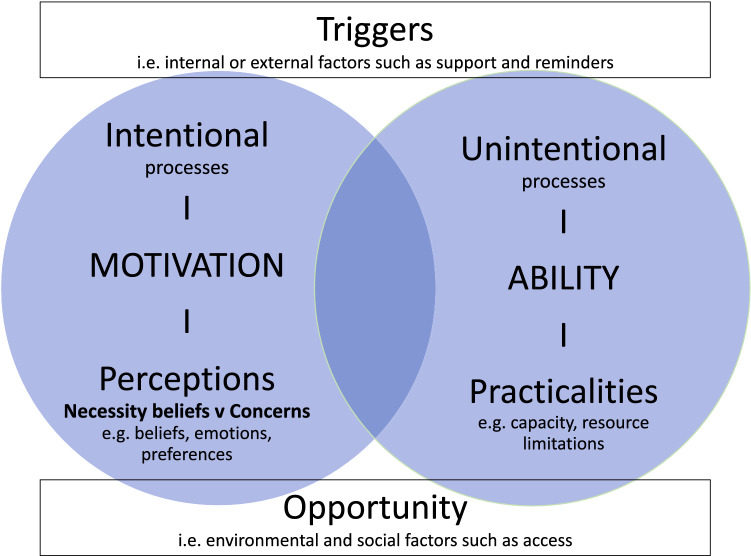
Perceptions and practicalities approach (including NCF). Adapted from Horne et al. [12]

From February 2014 to January 2016, the qualitative study team planned purposive sampling of up to 50 study participants to take part in semi-structured in-depth interviews (IDIs). Participants were interviewed from clinics in London, Sheffield, Manchester and Brighton. Up to the start of the PROUD study much of the topical and oral PrEP research on nonadherence had been collected in placebo-controlled trials and had reported unintentional reasons for nonadherence. As one of the first open label PrEP trials, the PROUD team used the Perceptions And Practicalities Approach as the guiding principle in designing the data collection tools and analysis frameworks (Fig. 1). To this purpose we aimed to distinguish the perceptual factors that inform intentional nonadherence such as motivation to use the product, from the practical factors that inform unintentional nonadherence such as capacity and resources.

They aimed to select 44 participants based on trial arm allocation (immediate or deferred), self-reported PrEP adherence among participants in the immediate group (high or medium/low) and changes in their self-reported sexual risk behaviour since baseline based on number of partners and condom use (increased risk or same/decreased risk). Adherence behaviour was consistently assessed based on self-reported adherence, clinic reported adherence, and pill counts. High adherence was defined as an average of 57% usage based on pill taking 4/7 days per week. From September 2015, they amended the selection criteria slightly in an attempt to identify more variability by risk behaviour, thereby selecting participants based on self-reported current risk behaviour (high risk or low/ medium risk based on highest quartile of partners and condomless sex and lowest quartile of partners and condomless sex). They aimed to purposefully select around six additional participants to investigate specific topics such as participants who had seroconverted while enrolled or had refused PrEP. Further details of this process are described elsewhere [25]. From February 2014 to January 2016, researchers who were independent of the study clinic team conducted the IDIs in English, lasting around 45–60 min using an in-depth interview guide designed with input from the social science advisory committee (Online Appendix 1). Participants provided written informed consent and were not compensated.

### Analysis

The IDIs were audio-recorded and then transcribed, coded and analysed in NVivo 11 using framework analysis [26]. We organised each interview into themes based on the topic areas on the IDI guide which were deductively identified from the literature and which related to the perceptions and practicalities likely to affect adherence including pill-taking routine and dosing schedule preferences. We coded the data within these themes and identified additional inductive themes that emerged from the data. After identifying text related to each theme, we agreed the analytical framework. The first author applied the analytical framework to the data creating a framework matrix in Microsoft Excel. Coding was checked against codes assigned to interview extracts by the last author. We then compared codes within and across participants to identify patterns and associations, particularly in relation to self-reported adherence.

The PROUD study protocol was approved by London Bridge Research Ethics Committee, the Medicines and Healthcare Products Regulatory Agency and each of the 12 participating Hospital Trusts. The trial is registered with ISRCTN (Number ISRCTN94465371) and ClinicalTrials.gov (NCT02065986). The study protocol, including participant information sheet (PIS) and informed consent form (ICF), and the in-depth interview PIS, ICF and interview guide, are available on the study website (www.proud.mrc.ac.uk).

## Results

In the results we first describe the participant characteristics. We then present results related to how participants reported using PrEP organized into four PAPA themes and 10 sub-themes that emerged from the data: (1) Reported adherence; (2) Perceptions (necessity beliefs, effectiveness, concerns); (3) Practicalities (daily routine, other daily medication, storage, alarms); and (4) Social and environmental factors (social support, mental illness, alcohol and drugs). We present quotations that exemplify the key findings and identify participants by selection criteria: trial arm, adherence and risk behaviour. We also state whether they had used PrEP by the time of the IDI, whether they enrolled in a London or out of London clinic, and their age-group at enrolment.

### Participant Characteristics

**Fig. 2:**
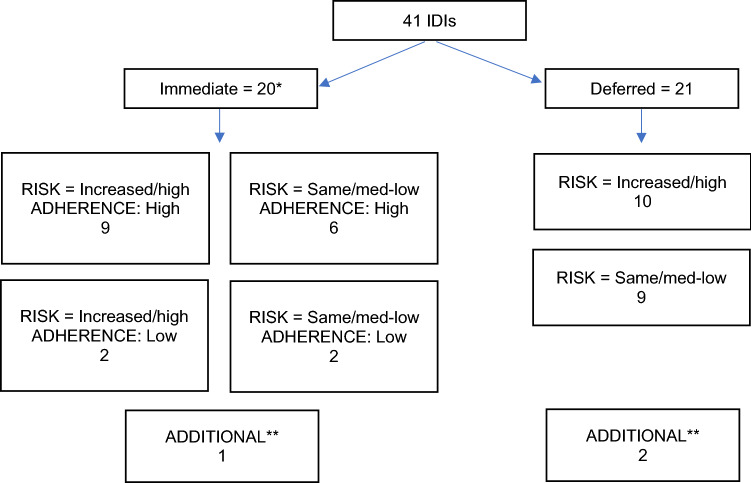
Purposive selection of participants for in-depth interviews based on trial arm allocation, risk behaviour and adherence

Forty-one PROUD participants were interviewed; thirty-eight selected equally from the immediate and deferred groups and three further IDIs with a trans woman, a participant who declined PrEP, and one who seroconverted during the trial (Fig. 2), (also reported in a qualitative study analysing the same interviews [25]).

Thirty-three participants said they were or had been using PrEP by the time of the interview. Thirty participants had been prescribed PrEP through PROUD, although two had discontinued it by the time of their IDI. A further three had accessed PrEP privately, one by using the Truvada from post-exposure prophylaxis (PEP) and another using Truvada from an HIV-positive partner (both during their deferred period of the trial), and a third who had purchased and taken it in the USA before joining the study. Adherence was high across the study, so it was difficult to select low adherers. When sampling, adherence was assessed on the basis of self-reported adherence, clinic reported adherence, and pill counts and high adherence was defined as an average of 57% or higher (4 out of 7 pills per week). Among those who we selected based on high adherence—their high adherence was confirmed in the qualitative IDI with just 5 people reporting low/medium adherence which was also confirmed during the IDI. Participants had used PrEP for a mean of 14.3 months by the time of the interview, ranging from one week to 32.8 months. The baseline demographic and sexual behaviour profiles of interviewed participants are shown in Tables 1 and 2 (also reported in a qualitative study analysing the same interviews [25]).

**Table 1:** Demographic and behaviour at enrolment

Median age	37.4
Interquartile range	(31.9, 42.7)
Clinic of enrolment	
London	24
Sheffield	9
Manchester	5
Brighton	3
Ethnicity	
White/Irish	34
Black, Asian and minority ethnic (BAME)^a^	7
Place of birth	
UK	26
Other^b^	15
University degree education	
Yes	25
No	16
Employed	
Yes	36
No	5
In a relationship	
Yes	17
No	24
Sexuality	
Gay	40
Bi-sexual	1
Gender	
Cis-male	40
Trans-female	1
Circumcised	
Yes	10
No	31
Symptoms of depression	
Yes	6
No	35
Chemsex used in the past 3 months	
Yes^c^	13
No	28
Post exposure prophylaxis (PEP) use in last year^d^	
Yes	14
No	26
Self-reported sexually transmitted infection (STI) in last year^e^	
Yes	18
No	21

^a^BAME ethnicities include Pakistani, Hispanic, Arabic, mixed ethnicity

^b^Other includes Australia, South America, South Africa, and the rest of Europe (1 missing)

^c^Chemsex use includes 12 participants using Gamma Hydroxybutyrate (GHB), 9 using methedrone, 7 using crystal meth

^d^PEP use excludes one missing response; six participants used PEP more than once

^e^STIs in last year excludes two missing responses

**Table 2:** Sexual behaviour in last 90 days, reported at enrolment

Median number of anal sex partners (interquartile range)	10 (3, 20)
Had anal sex with a new partner	
Yes	35
No	6
Been receptive partner	
Yes	39
No	2
Been receptive partner and condomless	
Yes	37
No	4
Been receptive during condomless sex with HIV positive man	
Yes	14
No	27
Been receptive during condomless sex with HIV positive man who you didn’t know was on treatment	
Yes	1
No	40
Been insertive partner	
Yes	40
No	1
Been insertive partner and condomless	
Yes	40
No	1
Been insertive during condomless sex with HIV positive man	
Yes	20
No	21
Been insertive during condomless sex with HIV positive man who you didn’t know was on treatment	
Yes	2
No	39

### Reported Adherence

In summary, most PrEP users said they rarely missed doses but did forget occasionally. A few said they missed a couple pills a month or that they missed less than a handful during the study. Eight participants said they never missed a dose.

I think I’ve maybe skipped, forgotten a dose twice—which I think in 2 years is pretty good” (Immediate/high-adherence/high-risk, on-PrEP, London, 40–45).

However, two participants had discontinued PrEP by the time of their IDI—one due to side effects and the other due to entering a monogamous relationship. Three other participants reported medium to low adherence prior to IDI selection. The one participant who we interviewed who seroconverted contracted HIV around two months after enrolling onto the deferred arm, and so never started taking PrEP.

There was no evidence of any clear patterns or associations between adherence and the demographics, location, relationship status or reported recreational drug use. However, this could be due to the small number of low adherers in our sample.

### Perceptions

Participants were probed on the perceptions and practicalities that impacted regular adherence. Below we discuss these perceptions such as their risk or need for PrEP, its level of effectiveness and concern about the potential negative impacts of taking PrEP (adverse impact on health, resistance to HIV or stigma).

### PrEP Necessity Beliefs

Participants in general reported being highly motivated to take PrEP with strong beliefs about the personal necessity for PrEP as a protection against HIV. Most participants recognised their own high-risk sexual behaviour and this was their main reason for joining the study.

Just one participant described intentionally missing days of PrEP due to feeling he did not need it. This participant described how his adherence was impacted when his perception of risk was low and outweighed by concerns around ‘unknown’ adverse effects:

I’ve taken this drug quite often and not having sex…I was thinking ‘do I really need to take this every single day?’ Because although I’ve no problem with it…there could be unknowns about it, so, at a particular time I decided not to and it had been absent from my bloodstream for ten days and then in fact I did have a high-risk sexual encounter…I learnt from that lesson that I should take it every day whether or not I think I’m going to have sex soon or not” (Immediate/high-adherence/low/medium-risk, on-PrEP, London, > 45).

This quote also highlights how participants may not always be able to predict when PrEP is needed.

As mentioned above, one participant decided to intentionally stop taking PrEP three months prior to the interview due to starting a monogamous relationship with an HIV-negative partner.

I came to the decision which made him very happy as obviously it also means that I am not sleeping around with lots of other guys…I just knew it was the right person” (Immediate/low-adherence/low-risk, stopped-PrEP, out-of-London, 40–45).

Four participants discussed periods of intentionally stopping PrEP for a short period due to health problems or illnesses when they felt at low or no risk of HIV due to reduced sexual activity, but this was always discussed with study staff.

### PrEP Effectiveness

When asked how effective they perceived PrEP to be, most accurately quoted figures of around 90%. Several people mentioned the caveat that good adherence was necessary to reach this protection, and this appears to have helped people commit to high adherence. Also, motivation to adhere may well have been impacted by the fact that the PROUD deferred arm was stopped early, in October 2014, due to early demonstration of PrEP effectiveness, and knowing these results could have reinforced good adherence after that time point. However, half of the interviews were conducted prior to October 2014 and participants reported similarly high adherence overall and most already understood PrEP to be highly effective. Those taking PrEP also understood that missing the occasional dose or taking one later than usual would not impact on the efficacy of PrEP.

### PrEP Concerns

#### Side Effects

Several participants reported concerns about side effects. Participants rarely intentionally missed doses due to side effects they experienced, but some expressed concern about potential and as yet unknown consequences of taking long-term medication. One participant did reduce their dose to every other day and subsequently stopped PrEP due to experiencing insomnia and neuropathy, which also caused him concern about the long-term consequences of PrEP. These side effects were discussed with the study staff, who agreed that in this instance stopping PrEP was the safest option.

“It switched every possibility of sleep off so to take it every day was not worth it but after a few weeks I went to taking it every other day…, then I got really strange neuropathy on my head and face and tingling and buzzing…so I tried stopping different things and…after a few weeks stopping the Truvada it sort of went into the background…” (Deferred/low/medium-adherence/low-risk, stopped-PrEP, London, 30–35).

Similarly, two participants declined PrEP due to, in part, concerns about the potential long-term side effects or feeling that it is unhealthy to take daily medication. Another participant was advised against PrEP by their doctor (outside the study) due to the potential adverse health impacts.

“It would be a good prevention if the pills weren’t so highly toxic”. (Purposively selected/declined-PrEP, deferred, London, 40–45).

#### Drug Resistance

In addition to concerns about side effects, concerns around resistance emerged several times, particularly when participants discussed taking PrEP on a long-term basis. However, these participants did not seem to understand the mechanism for resistant strains developing. For example, one participant felt he may not need PrEP anymore but that it would be risky to stop in case he wants to start again in the future and this process results in resistance, not understanding that resistance would only occur if he was HIV positive when restarting PrEP:

“I might want to come off it, I haven’t made the decision…because of the arguments you might build up some kind of resistance to it.” (Deferred/high-adherence/high-risk, on-PrEP, out-of-London, > 45).

Another participant in the deferred group was still undecided about whether to start PrEP due to his concern around resistance – particularly if he were to take a break during a monogamous relationship and then restart. One of the participants who declined PrEP due to concerns around side effects also worried that his body might get used to the medication in some way and then it would be ineffective as HIV medication if he were to become HIV positive.

#### Stigma

Overall there was little concern around stigma or negative perceptions of PrEP users. However, one participant in the deferred group planned on declining PrEP when offered because he was worried that other men would perceive him to be promiscuous and said he would have this perception of PrEP users himself.

“Also, I’m worried about the signals it sends, I’m single meeting people…they would think ‘why is this guy taking it?’ he must obviously be sleeping around…. personally, if I met someone doing it, I would probably think twice before I decide this is the right person…” (Deferred/decreased-risk, declined-PrEP, London, 30–35).

### Practicalities

Below we discuss practicalities that influence adherence such as establishing a routine versus an unexpected change in routine or the time of day at which the pills are taken, that can both aid and hinder adherence to daily PrEP. Other practical influencers include experience with other medication, pill storage, alarms, social support, mental health issues and alcohol and drug use.

#### Daily Routine

Inserting PrEP into other daily routines helped people remember, such as taking PrEP as soon as they wake up, when eating breakfast or brushing their teeth. Most participants reported taking PrEP every morning. Many said if they forgot in the morning, they could take it later that day. The only exceptions were two participants who took PrEP at midday and two others who chose to take it in the evening using an alarm to remember. Several participants, usually due to concern about side effects, initially took the pill in the evening, but found it was easier to forget then, so switched to the mornings. One such respondent with low/medium reported adherence said that he improved after deciding to take it in the morning with other daily medication.

“At the beginning, I took it in the evening…but what I found was because I wasn’t taking anything else in the evening—it was fairly frequent I forgot it, so in the end I realised I didn’t have side effects, I just threw it in with everything else” (Immediate/low/medium-adherence/low/medium-risk, on-PrEP, London, 30–35).

Similarly, another participant (quoted below) described struggling to remember to take the tablets at the start, although he also said he has since improved through establishing a morning routine, and in the last year has not missed more than one day in a row.

“It took me a while to get used to taking the pills on a daily basis…sometimes I would go three or four days without taking it” (Immediate/low-adherence/increased-risk, on-PrEP, out-of-London, 30–35).

The other main reasons for occasionally forgetting pills were disruptions to established routine. For example, unexpectedly staying away from home and leaving their pills at home.

“I do tend to miss two on average a month, and I think this month I missed two because I was away for two nights and because I’m so shit I forgot my tablets” (Deferred/high-adherence/medium-risk, on-PrEP, out-of-London, 25–30).

Two participants described hectic times at work that caused them to miss a dose and another described splitting up with his boyfriend and not wanting to return to his house to retrieve his PrEP.

#### Other Daily Medication

More than half of PrEP users had experience of taking other daily medications for chronic conditions, allergies or psychological disorders, or vitamins. Many explained how it is easy to integrate taking PrEP into this established routine.

“I take other medication on a regular basis so it just goes into the pillbox along with everything else. It must be harder for other people who don’t have to take medication on a regular basis” (Immediate/high-adherence/increased-risk, on-PrEP, London, > 40).

Some participants reported missing fewer doses since starting to take PrEP with other medication or said they now miss more PrEP after discontinuing other medication. Two participants also said they noticed symptoms such as wheezing or sneezing if they forgot their other medication and so this would remind them to take all their daily medication, including PrEP. In addition, most participants who were yet to start PrEP anticipated finding it easy to remember the pill due to already taking daily medication or vitamins.

#### Storage

Some clinics offered pillboxes and several participants found them helpful, particularly to check whether they had taken the dose already. Several already used one for other medication.

“At one point, I would set the timer and I would take it but then I wouldn’t remember whether I’d taken it, so I tried ticking it off. And then when I moved to the third clinic they gave me a box with Saturday to Friday written on it and it was dead easy after that” (Deferred/high-adherence/high-risk, on-PrEP, out-of-London, < 25).

Eleven participants described carrying spare pills with them or keeping pills in their gym-bag, car or at other people’s houses to avoid missing a dose. Three participants used a keychain holder because it was smaller and more discrete than a pill bottle.

“I always carried one in my car, so if I thought ‘oh damn I missed my tablet’ I could just go to my car at work and take the tablet” (Immediate/low-adherence/low-risk, stopped-PrEP, out-of-London, 40–45).

#### Alarms

Seven participants used a daily alarm or app and said this helped prevent them missing doses. Others said they have used them but don’t need alarms anymore. Two participants suggested that receiving a reminder every day or every week from the clinic would be helpful.

“To me it isn’t a problem, I just set a daily alarm on my phone with the label Truvada… I'm generally quite good at remembering anyway but if I do forget the alarm goes off and I take it there and then” (Deferred/decreased-risk, on-PrEP, out-of-London, 35–40).

### Social and Environmental Factors

#### Social Support

Most interviewees took their pills alone with minimal input from friends or family. A few participants actively wanted to avoid disclosing their PrEP use to people they lived with, although most did not express concern over this. Some participants discussed taking pills at the same time as their HIV-positive partner or being reminded to take PrEP by them.

“Usually I split my time between my house and my boyfriend’s house and he always carries spares for me in case I forget…I thought it would be really difficult because I’ve never managed to take medication but because I went on it…about 6 months after my boyfriend started taking his HIV medication, I went through the process with him of remembering to take it every day, and he was very good at reminding me to take it at the start” (Immediate/high-adherence/high-risk, on-PrEP, London, 30–35).

#### Mental Illness

One participant discussed forgetting his pills for a period of several weeks due to depression. Promisingly, they also said that the psychological support provided through the study was helpful.

#### Alcohol and Drugs

Drugs, chemsex and drinking were discussed frequently in terms of sexual risk behaviours and condom use, but rarely in relation to PrEP non-adherence. However, three PrEP users said they had forgotten a dose when hungover or drunk and one person missed doses when participating in chemsex. Conversely, an equal number of participants said that they make sure they remember even when drinking or taking drugs.

Participant: “Sometimes going on funny binges…I basically disappear for 3 or 5 days…”.

Interviewer: Would those binges impact on PrEP use?

Participant: “No, because I’m so organised that I actually have a bag and a few PrEP pills, I don’t think that’s ever caused me not to (take PrEP)” (Immediate/high-adherence/high-risk, on-PrEP, London, 40–45).

### PrEP Dosing and Administration Preferences

When asked about whether they would prefer an on-demand dosing schedule like the one used in IPERGAY, the majority of participants said they prefer the daily dose. Most said adherence would be difficult because it would be hard to predict when they are going to have sex and carrying the pills around would be a nuisance.

“If you plan to have all your sex in one week in one month and then do nothing—maybe I’ll be like that one day—but at the moment I don’t know if I’m going to have sex tonight or tomorrow or the next day until it happens” (Immediate/high-adherence/high-risk, on-PrEP, London, 30–35).

Participants often said it is easier to remember something every day because it becomes routine. Several felt a daily regimen is safer and one participant mentioned resistance may be more likely with non-daily schedules.

“…got a degree of safety net because if you miss one day it’s not the end of the world. But you also build it into a routine so it becomes an automatic rather than a you gotta think about this” (Deferred/increased-risk, on-PrEP, London, 30–35).

The only exceptions were the participant who discontinued PrEP due to experiencing side effects and two who said they would be interested in an on-demand schedule since they rarely have higher-risk sex.

Conversely, participants generally liked the idea of an injection every month or couple of months and saw this as easier than remembering pills. Significantly, two low adherers said they would prefer an injection, including the participant who struggled with adherence due to depression. Furthermore, two participants said they would prefer a monthly injection so they were not treated like or felt like they have a chronic disease.

“I would choose the injection—it would be less stressful. A sick person must remember to take pills every day, sometimes I feel like a sick person because I’m dependent on something every day. If I had the injection I wouldn’t feel like that” (Immediate/high-adherence/increased-risk, on-PrEP, London, 35–40).

The one participant who planned not to start PrEP when he was supposed to, discussed not liking the idea of taking pills for various reasons including inconvenience, being reminded of his behaviours, and the perception of others. He said an injection would therefore be preferable.

“I’m not always at home, I need to remember to pack the tablets in my bag, and it’s in your face, constantly being reminded that I’m living a risky lifestyle and I need to take medications to prevent myself from myself. Also, the perception of others around you they would see that you are taking a tablet if you are on holiday… with friends and they see you taking a tablet they might ask you what is that?—and if it was a vaccination in the privacy of a clinic no one would ever find out, and if you had a partner you were living with or you had children…you couldn’t keep it a secret” (Deferred/decreased-risk, declined-PrEP, London, 30–35).

## Discussion

In this analysis, we applied the perceptions and practicalities approach to explore factors that influence individuals’ motivation and ability to adhere to daily PrEP. This analysis demonstrates that the PAPA framework may be a useful approach for identifying potentially modifiable causes of non-adherence from the perspective of the individual and could inform future PrEP adherence support interventions [16].

Using the PAPA framework ensured we identified important perceptual, as well as practical, factors influencing adherence in this analysis. The key perceptions explored related to necessity beliefs and PrEP effectiveness. In this analysis participants had high perceived HIV risk and confidence in PrEP efficacy, two factors that clearly influenced the high level of adherence. Whereas, women’s low perception of HIV risk in the FemPrEP trial was used to partially explain the low levels of adherence observed, highlighting the importance of assessing individual necessity beliefs to optimise adherence [27]. Due to oral PrEP being so highly effective, doubts about efficacy are rarely seen in the literature as a barrier to adherence, although this was suggested in relation to earlier partially effective vaginal PrEP products [28]. As such the Necessity and Concerns Framework is a useful tool to understand adherence in different contexts as it posits that motivation to start and continue with a medication is influenced by the patient’s beliefs and how they judge their personal need for treatment relative to their concerns about taking it.

Our findings also emphasised the importance of PrEP concerns in relation to adherence. Interestingly, concerns about PrEP went beyond side effects and included beliefs about long term effects and perceptions of stigma associated with treatment. PROUD participants perceived concerns about drug resistance were based on misunderstandings about the mechanism of resistance, an issue that clearly needs to be further clarified in health promotion materials for PrEP. As was the case in PROUD, concern over potential side effects was a perceived factor inhibiting uptake for a minority of PrEP naïve MSM in Boston [29]. Concerns around side effects were also a barrier to uptake for most MSM who declined PrEP in a study in China, and were the main reason for discontinuing PrEP for half of those who stopped taking it [30]. As PrEP is rolled out globally to all at risk populations, it will be important to monitor the types of concerns reported and the ways in which these concerns influence adherence as the types and level of concern around PrEP may vary across cultures and geographies in relation to differing social norms and health seeking behaviours.

In PROUD, only one participant declined the offer of PrEP due to concerns about PrEP-related stigma but stigma was rarely discussed by those taking PrEP. Whereas, stigma associated with taking PrEP, either due to others perceiving a user to be HIV-positive, being sexually ‘promiscuous’ or due to homophobia are frequently discussed as negatively influencing adherence in the literature [29–37]. HIV stigma is also known to impede ART adherence [38]. The PROUD study clinics were based in large English cities with well-established gay communities. However, concerns about stigma in other areas may be different and interviews with MSM in Scotland found some would be concerned about their roommates or family seeing PrEP tablets and mistaking them for HIV medication [22]. The PrEP Impact Trial results may provide additional evidence of the role PrEP-related stigma has on adherence for other at risk populations such as cis women, cis men having sex with women, trans women, trans men, and non-binary individuals. [39]. While this data may also provide insights into the influence of PrEP-related stigma among sex workers, additional research is needed to understand its influence among other potential PrEP users such as people who inject drugs, people who are incarcerated and people who are homeless.

Practicalities that positively influenced adherence were consistent with multiple studies of PrEP adherence [29, 32–34, 36]. In various settings, experience with daily medications and vitamins help people to adhere to daily PrEP [29, 30, 34, 36]. Similarly, TGW in Thailand mentioned integrating PrEP use with their hormone therapies positively influenced adherence [36]. These findings suggest that patients without medication experience may need additional adherence support such as pill boxes and alarms [34].

Our findings were also consistent with the notion that environmental factors and the social context are important determinants of adherence acting on both perceptions and practicalities. Disclosure to a partner has been shown elsewhere to be an important practical factor in supporting adherence. A quantitative analysis of PrEP adherence during the iPrEx open-label extension study found that those who disclosed their study participation to their partner were 15% more likely to have protective blood concentrations than those who did not [40]. Other practical factors such as travel, disruptions to routine and busy periods were common reasons for non-adherence. These factors were also identified in previous qualitative studies examining PrEP adherence amongst MSM, for example in a phase I trial in Kenya and the iPrEx trial in Thailand, where travelling was a leading cause of participants missing several consecutive doses [32, 34, 36].

Some PROUD participants discussed drugs and alcohol as influencers of higher risk sexual behaviour. However, several describe making sure to take PrEP even when using substances and did not report substance use as a negative influence on PrEP adherence. This finding is consistent with a quantitative analysis that examined the relationship between adherence and chemsex during the PROUD trial [41]. Similarly, a qualitative study with substance users in San Francisco noted that no one reported fewer than four doses per week and so this population can adhere to and benefit from PrEP [33]. Conversely, periods of mental ill-health are a potential negative influencer for some people, which other qualitative studies have also found [29, 34].

PROUD participants, who mainly perceived themselves as at regular high-risk of HIV, preferred daily dosing of PrEP (when provided for free at least) over on-demand regimens. However, other oral dosing options and longer-term injectable options have the potential to overcome some of the perceptual and practical factors that inhibit uptake and adherence for some people. It is possible that those taking PrEP were biased in this opinion by already practicing and getting used to a daily regimen, although many stated a preference for daily over on-demand dosing due to their inability to predict sexual encounters. Other studies investigating on-demand dosing among MSM highlight barriers to adhering to this dosing scheduled including unanticipated sexual encounters, taking drugs or alcohol around the time of sex, falling asleep, and concern about taking PrEP in front of a sexual partner because it causes suspicion around one’s HIV status, loyalty or promiscuity [31, 32, 42]. Indeed, quantitative analyses of trials comparing both schedules find lower reported adherence to non-daily compared to daily regimes [43–45]. However, some benefits of non-daily dosing may include lower cost, fewer doses and less severe side effects, which may be seen as less burdensome [32, 46].

Many PROUD participants felt that a long-acting injectable form of PrEP would be most convenient, especially for those who struggle with adherence. This was also expressed during FGDs with MSM and trans men and women in the U.S. [47] In another study in the U.S, a third of oral-PrEP users reported a preference for long-acting injectable PrEP although respondents who expressed concerns about the reliability of consistent efficacy were less likely to report willingness to use them [48]. Long acting Cabotegravir injectable PrEP has been shown to be highly effective in preventing HIV in MSM and TGW and has been approved by the US Food and Drug Administration (FDA) [49]. Additionally year-long implants are in preclinical phases of research [50, 51]. These are both exciting potential forms of PrEP, particularly for those who struggle with adherence or do not wish to take daily medication.

One strength of the study is that due to the PROUD trial we were able for the first time in England to conduct interviews with PrEP users, rather than people discussing hypothetical or anticipated pill-use. Using the PAPA framework ensured we identified important perceptual factors influencing adherence, which have typically received less attention in the HIV prevention adherence literature than practical factors.

A limitation of this study is the small number of participants interviewed who had low adherence. This makes it difficult to identify common themes that influence non-adherence or compare other associations such as with trial arm, demographics or their partner’s status. Also, as mentioned, the interviews were conducted in the context of a daily PrEP protocol, with non-daily PrEP use only discussed after the release of the IPERGAY trial results [3]. Therefore, this context will have influenced participants’ preferences and perceptions of how their behaviours or adherence might change on a different schedule. Similarly, due to the duration of follow-up, we did not interview participants who were using PrEP seasonally, during higher periods of sexual risk behaivour. Understanding the cyclical nature of PrEP use is going to be vital and as argued elsewhere, it is necessary to move beyond a binary assessment of PrEP use [42]. Additionally, TGW are underrepresented in the literature but are disproportionately impacted by HIV [52]. In the PROUD cohort, only three TGW enrolled in the trial and only one trans woman was interviewed. Therefore, we could not disaggregate the findings for TGW. Also, Black Asian and minority ethnic GBMSM and people from lower socio-economic groups were underrepresented in the PROUD interviews, meaning findings may be biased towards the views and experiences of this mainly white, and higher socio-economic demographic. Due to the novel nature of PrEP at the time of PROUD, this cohort of participants were highly motivated to use PrEP and therefore reported few structural, social and environment factors that inhibited adherence. It will be important to probe these factors further in implementation programmes. Finally, the interviews may be biased towards participants who are willing to invest additional time and effort into PROUD, since the IDIs were conducted outside of study procedures.

### Recommendations

Carrying out a non-judgmental assessment of adherence with PrEP users and offering tailored adherence support is a crucial part of PrEP provision and should be continued outside of clinical trials, as recommended by WHO and NHS England and Scotland [53–55]. Based on our analysis, we recommend further application of the PAPA framework to understand perceptual and practical factors that influence individual PrEP adherence and the adaptation of the NCF to PrEP to inform the best way to support individual adherence over time.

Perceptual factors are the key influencers of uptake and adherence and may be addressed by improving communication between provider and patient [11]. Therefore, addressing concerns related pill-use such as side effects when promoting and discussing PrEP initiation is crucial. Our findings suggest the risk of drug resistance also needs clear explanation to avoid confusion and concern. Motivational interviewing is one technique recommended by the WHO for PrEP counsellors which could be used to explore people’s perceptions of PrEP and foster their motivations to adhere [54]. Also, as PrEP is rolled out to a wider, more diverse group of GBMSM in the UK, social perceptions of PrEP may challenge adherence. More research is needed on how best to help GBMSM avoid or deal with these negative reactions to PrEP use.

These findings and others [29, 34, 36] suggest adherence counselling should include tools and techniques based on the practical facilitators discussed (such as pill boxes, carrying spare pills, alarms, text reminders and incorporating PrEP into one’s daily routine or established pill-taking routine) to overcome issues of non-adherence. We recommend discussing these strategies with PrEP users to build on existing habits to create a personalised PrEP routine. This could include recommending disclosing PrEP use to a partner or trusted person [54], or involving partners in PrEP adherence interventions in the future [40]. Initial learnings from the NHS Scotland PrEP programme suggests that both daily and on-demand schedules are popular and that many people will switch between dosing regimens [53]. Therefore, building skills around on-demand schedules, and cyclical seasonal use of PrEP, is an important area for further research. The PrEP Impact Trial results will help inform the feasibility of tailoring the dosing regimen depending on the individual’s needs and desires at different times during their PrEP use [39]. Now that PrEP is available on the NHS, it will be vital to continue to monitor the ways in which structural, social and environment factors influence perceptions and practicalities of PrEP uptake and adherence [56].

## Conclusion

In conclusion, gay, bisexual and other MSM in this study were highly motivated to use PrEP by the perception of their high risk of HIV and high levels of PrEP efficacy, and therefore reinforcing the *necessity* for PrEP. Also in line with NCF, it appears that concerns about side effects and resistance, and potentially stigma, can influence PrEP initiation and adherence although rarely outweighed necessity among this cohort. The NCF assessment, once adapted for PrEP, could help tailor adherence support for PrEP users. Practical factors that influenced non-adherence to daily PrEP use include travel, schedule disruptions and, less commonly, mental health issues, although in this cohort substance use did not appear to impede adherence. These factors can be largely overcome with adherence support including practical tools and social support. Communicating such strategies should remain an integral part of PrEP provision and support in clinical settings. Finally, this generally high risk and highly adherent cohort viewed a daily oral dose as easier to adhere to than on-demand dosing, but showed interest in long-acting injectable options. However, non-daily oral schedules will still be preferable to some GBMSM. Supporting PrEP adherence will remain an important public health challenge as more people take up different dosing regimens of PrEP in clinical settings in the UK and elsewhere.

## Supplementary Information

Below is the link to the electronic supplementary material.

Supplementary file1 (DOC 54 KB)

## Data Availability

The PROUD data are held at MRC CTU at UCL, which encourages optimal use of data by employing a controlled access approach to data sharing (http://www.ctu.mrc.ac.uk/our_research/datasharing/). All requests for data are considered and can be initiated by contacting mrcctu.ctuenquiries@ucl.ac.uk or through the URL: http://www.ctu.mrc.ac.uk/our_research/datasharing/application_process/. The basis for this project originated from a MSc research project undertaken by DAF at the London School of Hygiene & Tropical Medicine (LSHTM), and ethical approval for this MSc project was granted by LSHTM. Data was accessed from University College London (UCL) and the Medical Research Council (MRC) Clinical Trials Unit (CTU) through a clinical data disclosure agreement and PROUD sub-study proposal agreement.
