# Supplementary material for: Perceptions and Practicalities Influencing Pre-exposure Prophylaxis Adherence Among Men Who Have Sex with Men in England
Source: AIDS Behav. 2022 Feb 19;26(8):2768–82. doi: 10.1007/s10461-022-03624-6 (PMC9252952; doi:10.1007/s10461-022-03624-6)
Supplement: Supplementary file 1 — Supplementary file1 (DOC 54 KB) [file 10461_2022_3624_MOESM1_ESM.doc]

**PROUD Pilot Study: Guide for one-to-one discussion**

**INSTRUCTIONS FOR INTERVIEWER:**

- Discuss the supplementary participant information sheet with the participant and complete the informed consent form.
- The guide includes 5 topics for discussion – with possible probes in *italics*. All topics should be covered with men in the immediate start group who are using Truvada, as well as men in the deferred start group who are not yet on Truvada [wording changes indicated in brackets].

**Discussion topics:**

1. What do you think about PrEP?
   1. *For you, for your sexual partner(s), for others*
   2. *Pros/cons of PrEP – including level of effectiveness*
2. What do you think about the PROUD study?
   1. *Why did/would/wouldn’t you, your sexual partner(s), other gay men take part?*
   2. *Pros/cons of PROUD – including does study meet expectations*
3. How does [do you think] being on PrEP [will] affect the way you see yourself? And the way others see you?
   1. *How, what and with whom have you communicated about PrEP?*

**Permissive statement:** I would now like to discuss drug adherence. We understand that many people on preventative medication find it very difficult to take it regularly and often miss doses. It is common for people not to take medication perfectly. Although daily use is the ideal option, in this study we are particularly interested in finding out how PrEP is (will be) taken realistically.

1. How do you [do you think you will] use PrEP?
   1. *Barriers and facilitators, reasons for patterns of use*
   2. *Recommendations on future provision of PrEP*
2. How does being part of this study influence how you feel about your sex life?
   1. *How does/will being on PrEP influence how you feel about your sex life?*
   2. *Changes in behaviour, risk perception, risk reduction*

**Closing statement:** Before we end, are there any observations or recommendations for the research team that you would like to offer that have not been discussed?

**INSTRUCTIONS FOR INTERVIEWER:**

- Address any questions and thank participant for attendance

**Potential discussion points for PROUD study IDI**

| 1. **What do you think about PrEP**   *For you, partners, others; pros & cons* | | 1. **Why did you take part in the study**   *Pros & cons* | |
| --- | --- | --- | --- |
| - Benefits: additional/ alternative prevention, risk management - Downsides: side-effects, resistance, risk compensation - Awareness pre-study | - Effectiveness: knowledge/belief of - Relative effectiveness v other risk reduction - Previous experience of PEP/ PrEP | - Motivation/ barriers - Expectations v reality - Comprehension of study aims/design/ risks/benefits - Randomisation: understanding/ acceptability of | - Acceptability of study tools/ requirements - Intention to remain in follow-up – reason would leave - Most like/ dislike |
| 1. **How does (will) being on PrEP affect the way you see yourself & others see you**   *How/what/whom talked about PrEP* | | 1. **How do you (will you) use PrEP**   *Barriers/facilitators/reasons for patterns; Recommendations for future provision* | |
| - Individual attitudes to risk perception/ taking/ reduction - Societal attitudes to risk perception/ taking/ reduction /PrEP - Ideal PrEP user / inference of using PrEP | - Discussions about PrEP and reactions: partners/web-dates/ friends/ family - Reasons for not discussing with all/some people - Most like/dislike about way PrEP makes you/others see you | - Use patterns – timing (day/hour), in relation to sex, storage, reminders, support - Experience with other daily medication - Example of last day taken/ not taken - Perceived effectiveness of use patterns – better with more, manage with less | - Response to sex when not taken - Use in relation to other risk reduction - Ways to enhance adherence - Practicality of long term v periods of daily use - Dosing preferences - Recommendations on how to provide PrEP in future |
| 1. **How does being part of this study influence how you feel about your sex life**   *Changes in behaviour, risk perception, risk reduction* | | 1. **Additional** | |
| - Influence of recording behaviour, counselling, HIV/STI testing, PrEP - Influence on risk taking, sex regret, confidence, intimacy, arousal, satisfaction - Changes in: partners (no., type, places), sex frequency/type (non-anal), condoms, sero-sorting, positioning | - Perception of, and response to, safer/ less-safe sex - Influence of drink/drugs - Usual /ideal sex - Increase/decrease of control of sex/risk - Pressure/ violence - Changes for better/ worse - Expectation of STI/HIV infection | - Feasibility of trial - Cost-effectiveness of PrEP (£5k pa) - Acceptability of PK tests | - Feedback on experience/ convenience of IDI - Feedback to study team - If could change one thing about study, what would it be? |
